# Huxley's Way of Life

**Published:** 1888-03-03

**Authors:** 


### The Hospital

A Weekly Institutional Journal of

**Science, Medicine, and Philanthropy.**

*For Hospitals, Asylums, Medical Practitioners, Students, Nurses, Families, Parishes, Congregations, and the Charitable Public.*

Vol. III. No. 75.] [March 3, 1888.

### Huxley's "Way of Life."

A bold reformer, a believing evangelist, and a wise teacher of an old and new gospel is Professor Huxley. Like other apostles of some other faiths, the zealous propagandist mixes up much matter with the prime articles of his creed which we can by no means agree with, and which, we think, might be successfully controverted were this the place and time. But with his fundamental position on the question of practical instruction for practical life we entirely agree. It makes a man both sorry and angry to see that so obvious, so useful, so indispensable, and so easily understood a gospel as that which Professor Huxley preaches in the *Nineteenth Century* for February is listened to by nine-tenths of his countrymen with about the same amount of attention as they pay to an announcement that Lieutenant Jones was presented at Court yesterday, and that he wore exceedingly square-toed boots. By the thinking and doing few everything he says is held to be worthy of consideration—consideration it may be to confirm, or consideration not seldom to confute. By the many he is often regarded—as John the Baptist (whom he is not altogether unlike) complained that he was—as a mere "voice crying in the wilderness."

Professor Huxley is known by all men to be an accomplished man of science and a powerful and skilful wielder of the pen. He can, and does, fight in the very best and most approved British style, openly and fairly, but also sternly, unflinchingly, and to the death. No mere reader or dabbler ever shows such grit and determination as this redoubtable scientist and sociologist; and it must be a grievous disappointment and disgust to him to find his laboriously quarried, painfully hewn, and honestly constructed work passed by as if it were only so much lath and plaster and stucco, instead of a permanent structure, built of the solidest stone. The unthinking many do not know the difference between a Huxley and a Hahnemann, an Ivanhoe and a Don Quixote. Hahnemann was a "man of science," and so is Huxley; Wilfrid of Ivanhoe was a knight, and Don Quixote was a knight too.

Poor Carlyle longed to be taken as a practical man—ardently desired above all things to see his principles built up into concrete and visible realities. Huxley, like every other real human being, shows in every page of his work his intense earnestness for results—visible, tangible, useful, enduring results. Results cannot but spring from his work as corn must grow from seed; only the December is often very cold, and the wheat lies long in the ground, and sometimes the sower himself never sees the grain in its full golden prime.

The Professor disclaims any relation to philanthropy. He is not a philanthropist, he says. But what is a philanthropist? Is it an animal that fusses in the newspaper, talks on platforms, puts its name at the head of well-known subscription lists, gives cheap tea and bad buns to the Sunday-school children, and otherwise makes itself unpleasant to persons of severe Arnoldian taste? It may be! But just as there are dogs and dogs, so there are philanthropists and philanthropists; and it would not be difficult to construct a correct definition of philanthropy which should include Professor Huxley within its all-embracing range.

In the article under consideration, "The Struggle for Existence," is the burden of the prophet's message. The topic, one might have thought, was stale; but no topic is stale unless the man who deals with it is stale. The teacher uses it like a man who has struggled, and still is struggling, to exist; to exist not merely as an animal, but as a man—an intellectual, moral, backward and forward looking man. Strange thoughts Huxley wrestles with; strange visions he makes to pass before our eyes! But these thoughts are not strange in the sense of being frivolous and fleeting, like soap bubbles. They are strange in the sense of being dark mysteries and profound problems such as are only known to exist by those who gaze steadfastly with open eyes into the facts and reasonings which constitute the universe and man. But though, like a true man of science, he follows his work intellectually into every region which his powers enable him to breathe and live in, he does not forsake and despise the easier problems which are common to him with the rest of mankind. Rather he comes down from the table-land of high speculation to the lower levels where other men are struggling, toiling, and fighting below; and, taking up the tools of peaceful labour or the weapons of unavoidable war, he shows those who are less competent than himself how these should be handled in order to win the greatest possible measure of success in calm or storm. Is that philanthropy? Little does it matter by what name it may be called! It is high service for mankind; and all men who have sense and conscience will do well to profit by it.

A greater teacher than Huxley has said that all his gospel may be condensed into two commandments, "Thou shalt love the Lord thy God with all thine heart; and thy neighbour as thyself." Huxley's gospel of social well-being may be reduced to one principle—the principle of "salvation by works" as he expresses it. "Our produce," he insists, "must be better and cheaper than that of other people"; and in order to this we Britons of all classes must live on good terms with each other, so that our huge workshop of a country may with most advantage devote itself to the turning out of the highest products of skill. *Good work* and *social stability*—these are fundamental articles of the Professor's sociological faith. His gospel is in a sense as old as history, or even as old as man. In the most savage period he who devoted himself with greatest thoroughness to his work usually commanded the best results. There is a measure of newness in it, however; and that newness consists in this—that men should *unite* to struggle, instead of dividing. They should struggle side by side against common difficulties and common enemies, instead of struggling against these difficulties and foes, and against each other as well. But even this is only new in the sense that it is newly apprehended, and propounded from a new standpoint, by a man of science. As a principle applicable to tribes it is at least as old as Moses; and as applicable, not only to nations, but to all the world, it is as old as Christ and Christianity. It can be proved by a thousand references that Christ taught all men the inestimable advantages of brotherhood, united action, thoroughness, and self-denial for the common good.

Into the details of Professor Huxley's programme we need not enter; those who wish may study them for themselves in the pages of the *Nineteenth Century* for February. Everyone who desires, either as a philanthropist or as a patriot, to promote the true and permanent well-being of all classes should hold it a serious duty to acquire the fullest possible knowledge and the clearest understanding of the questions which are now pressing for consideration, and of the means proposed for their solution. There are thirty-six millions of people in these islands; we do not produce food for more than eighteen millions. Many of these are in a state of starvation; many more are on its borders; many have no work and no hope of any. Whoever possesses intelligence and conscience should turn his thoughts to this subject. He cannot begin better than by taking his first lesson with Professor Huxley.

